# Quantitative Liver MRI Combining Phase Contrast Imaging, Elastography, and DWI: Assessment of Reproducibility and Postprandial Effect at 3.0 T

**DOI:** 10.1371/journal.pone.0097355

**Published:** 2014-05-19

**Authors:** Guido H. Jajamovich, Hadrien Dyvorne, Claudia Donnerhack, Bachir Taouli

**Affiliations:** Translational and Molecular Imaging Institute, Department of Radiology, Icahn School of Medicine at Mount Sinai, New York, New York, United States; Institute of Psychology, Chinese Academy of Sciences, China

## Abstract

**Purpose:**

To quantify short-term reproducibility (in fasting conditions) and postprandial changes after a meal in portal vein (PV) flow parameters measured with phase contrast (PC) imaging, liver diffusion parameters measured with multiple b value diffusion-weighted imaging (DWI) and liver stiffness (LS) measured with MR elastography (MRE) in healthy volunteers and patients with liver disease at 3.0 T.

**Materials and Methods:**

In this IRB–approved prospective study, 30 subjects (11 healthy volunteers and 19 liver disease patients; 23 males, 7 females; mean age 46.5 y) were enrolled. Imaging included 2D PC imaging, multiple b value DWI and MRE. Subjects were initially scanned twice in fasting state to assess short-term parameter reproducibility, and then scanned 20 min. after a liquid meal. PV flow/velocity, LS, liver true diffusion coefficient (D), pseudodiffusion coefficient (D*), perfusion fraction (PF) and apparent diffusion coefficient (ADC) were measured in fasting and postprandial conditions. Short-term reproducibility was assessed in fasting conditions by measuring coefficients of variation (CV) and Bland-Altman limits of agreement. Differences in MR metrics before and after caloric intake and between healthy volunteers and liver disease patients were assessed.

**Results:**

PV flow parameters, D, ADC and LS showed good to excellent short-term reproducibility in fasting state (CV <16%), while PF and D* showed acceptable and poor reproducibility (CV = 20.4% and 51.6%, respectively). PV flow parameters and LS were significantly higher (p<0.04) in postprandial state while liver diffusion parameters showed no significant change (p>0.2). LS was significantly higher in liver disease patients compared to healthy volunteers both in fasting and postprandial conditions (p<0.001). Changes in LS were significantly correlated with changes in PV flow (Spearman rho = 0.48, p = 0.013).

**Conclusions:**

Caloric intake had no/minimal/large impact on diffusion/stiffness/portal vein flow, respectively. PC MRI and MRE but not DWI should be performed in controlled fasting state.

## Introduction

Several non-invasive imaging techniques have been recently developed for detection of liver fibrosis and portal hypertension with variable success rates. These include ultrasound based techniques such as transient elastography (Fibroscan) [1]–[6]; and MRI techniques such as MR elastography (MRE) [7]–[10], diffusion-weighted imaging (DWI) [10]–[15], and dynamic contrast-enhanced (DCE) MRI [16]–[19].

Some of the parameters extracted from these techniques measure hepatic flow, which may vary depending on the prandial state as the blood flow into the splanchnic circulation increases after a meal, with an increase in portal vein (PV) flow as measured with Doppler ultrasound [20]–[26].

Liver apparent diffusion coefficient (ADC) measured with DWI has been shown to negatively correlate with the stage of hepatic fibrosis [11], [27], [28]. ADC is influenced by several factors, including T2, perfusion, cellularity [29], and possibly prandial state [30]. Intravoxel incoherent motion (IVIM) DWI acquisition can be used to separate true diffusion from perfusion by computing a true diffusion coefficient (D or Dt), a pseudo-diffusion coefficient (D*) and the fraction of flowing blood (perfusion fraction: PF or f) [31]. These parameters have also shown potential to diagnose fibrosis and cirrhosis [15], [19], [32]. There is currently no published study assessing the changes in IVIM parameters after caloric intake.

MRE consists of imaging shear waves propagating through tissue in order to assess tissue stiffness [33]. MRE has been shown to be highly reproducible [34]–[36] and has been used as a noninvasive technique to detect liver fibrosis [7], [37]–[39]. It has recently been suggested that liver stiffness (LS) measured with MRE or transient elastography changes after a meal [26], [40].

Phase contrast (PC) MRI is a method that can be used to measure PV flow with a lower variability and higher reproducibility than Doppler ultrasound [41]–[43]. The portal flow dynamics change with progression of chronic liver disease and its measurement may be of value for diagnosis and evaluation of the severity of portal hypertension [23], [44]. PV flow measured with PC-MRI was shown to change significantly after caloric intake [30], [45], [46].

The objective of this study was to quantify PV flow parameters using PC MRI, liver diffusion parameters using IVIM DWI, and LS using MRE in fasting and non-fasting conditions, in healthy volunteers and patients with liver disease. A secondary objective was to assess the short-term reproducibility of these parameters and to compare these metrics in healthy volunteers vs. patients with liver disease.

## Materials and Methods

### Subjects

This HIPAA compliant prospective single center study was approved by the Icahn School of Medicine at Mount Sinai Program for the Protection of Human Subjects. Written consent was obtained from all subjects. 11 healthy volunteers (6 males, 5 females; mean age 30.6 y) and 19 patients (17 males, 2 females; mean age 55.8 y) with chronic hepatitis C virus infection were enrolled in this study from July 2012 to December 2013. Volunteers were considered healthy if they had no history of liver disease or significant alcohol consumption. Patients were enrolled in the study if they had a liver biopsy performed within 3 months of the MRI study or were diagnosed with liver cirrhosis based on imaging findings. Subjects were not considered for this study if they had diabetes.

### MRI Acquisition

**Figure 1:**
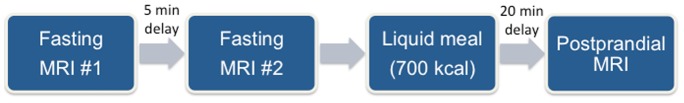
Study design. All subjects were scanned twice after 6 hours of fasting to assess short-term reproducibility of MRI metrics (subjects were removed from the MRI system and re-imaged). Subjects were then scanned again in postprandial conditions, 20 min. after a 700 kcal liquid meal.

All subjects underwent three MRI exams on the same day using a 3.0 T multichannel system (MR 750, GE Healthcare) with a 32-channel phased-array torso coil. Subjects were initially scanned twice after 6 hours of fasting to assess short-term reproducibility of MRI metrics; subjects were first imaged and then taken off the scanner table (with coils removed and plugged back) and then re-imaged after a 5 min. break. Subjects were subsequently asked to drink a liquid meal of 700 kcal (Ensure, Abbott Nutrition) outside the MRI room **(** **Fig. 1** **)**, and were then scanned for a 3^rd^ time 20 min. after the end of the meal.

All subjects underwent the following sequences **(** **Table 1** **)**:

**Table 1:** Sequence parameters of the cine phase contrast, DWI and MR elastography (MRE) sequences obtained at 3.0 T in fasting and postprandial states.

	Phase contrast imaging	DWI	MRE
**Motion control**	Breath-hold with cardiac triggering	Free breathing	Breath-hold
**Sequence type**	2D GRE	2D SE SS EPI	2D GRE
**Orientation**	Perpendicular to portal vein	Transverse	Transverse
**TR (ms)**	6.1	3000	50
**TE (ms)**	3.3	52.5	22
**Flip Angle**	20^o^	90–180^o^	20^o^
**FOV (mm)**	380×304	435×435	360×360
**Image matrix**	192×160	128×128	128×80
**Slice thickness (mm)**	7	8	10
**Number of slices**	1	20	4
**Acceleration**	ASSET R = 2	ASSET R = 2	ASSET R = 2
**Acquisition time**	15–20 s	3:55 min	68 s

- Coronal and axial breath-hold SS FSE T2 (for anatomical purposes).
- 2D cine phase contrast (PC) imaging: using a breath-hold 2D GRE sequence with pulse triggering. 25 images were acquired spanning the cardiac cycle, with an encoding velocity of V_ENC_ = 50 cm/s for the PV. The acquisition plane was perpendicular to the extrahepatic PV (based on coronal SS FSE T2-weighted images). This sequence generated a phase image where each pixel value is proportional to the velocity through the imaging plane.
- IVIM DWI: using free breathing fat suppressed SS EPI DWI sequence sampling 16 b values (0, 15, 30, 45, 60, 75, 90, 105, 120, 135, 150, 175, 200, 400, 600 and 800 mm^2^/s), in axial orientation, covering the whole liver. The number and distribution of b values were chosen such that both pseudo-diffusion decay (b ≤100 s/mm^2^) and molecular diffusion decay (b ≥200 s/mm^2^) could be characterized with high stability and accuracy [15].
- MRE: A 19 cm diameter, 1.5 cm thick acoustic pressure-activated passive driver was placed in the right anterior chest wall centered at the level of the xiphoid process. The driver generated 60 Hz shear waves in the liver. Four axial slices (in 4 breath-holds) were acquired in the liver with a gradient echo multi-slice 2D sequence. A 2D multi-scale direct inversion algorithm generated LS maps from wave images [33].
- No contrast injection was performed as part of the study.

By starting the postprandial exam with a delay of 20 min, LS was re-measured approximately 30 minutes after the caloric intake, which corresponds with the maximum changes in splanchnic hemodynamics postprandially for cirrhotic patients [47]. In addition, for the first 19 subjects participating in the study (10 volunteers and 9 patients), the PV flow was again measured after acquiring LS. The PV flow was not significantly different when measured at 20 minutes vs. 30 minutes (p = 0.24, paired Wilcoxon test).

### Image Analysis

Images were processed by observer 1 (GHJ, a post-doctoral fellow with 2 years’ experience in image analysis). Regions of Interest (ROIs) were placed with supervision by a body MR radiologist (BT) with 10 years’ experience using 3 initial test cases. Measurement included the two fasting MRI studies and the postprandial study. ROIs were placed in the same locations between fasting and postprandial studies, and between repeat fasting studies, as follows.

#### PC MRI

**Figure 2:**
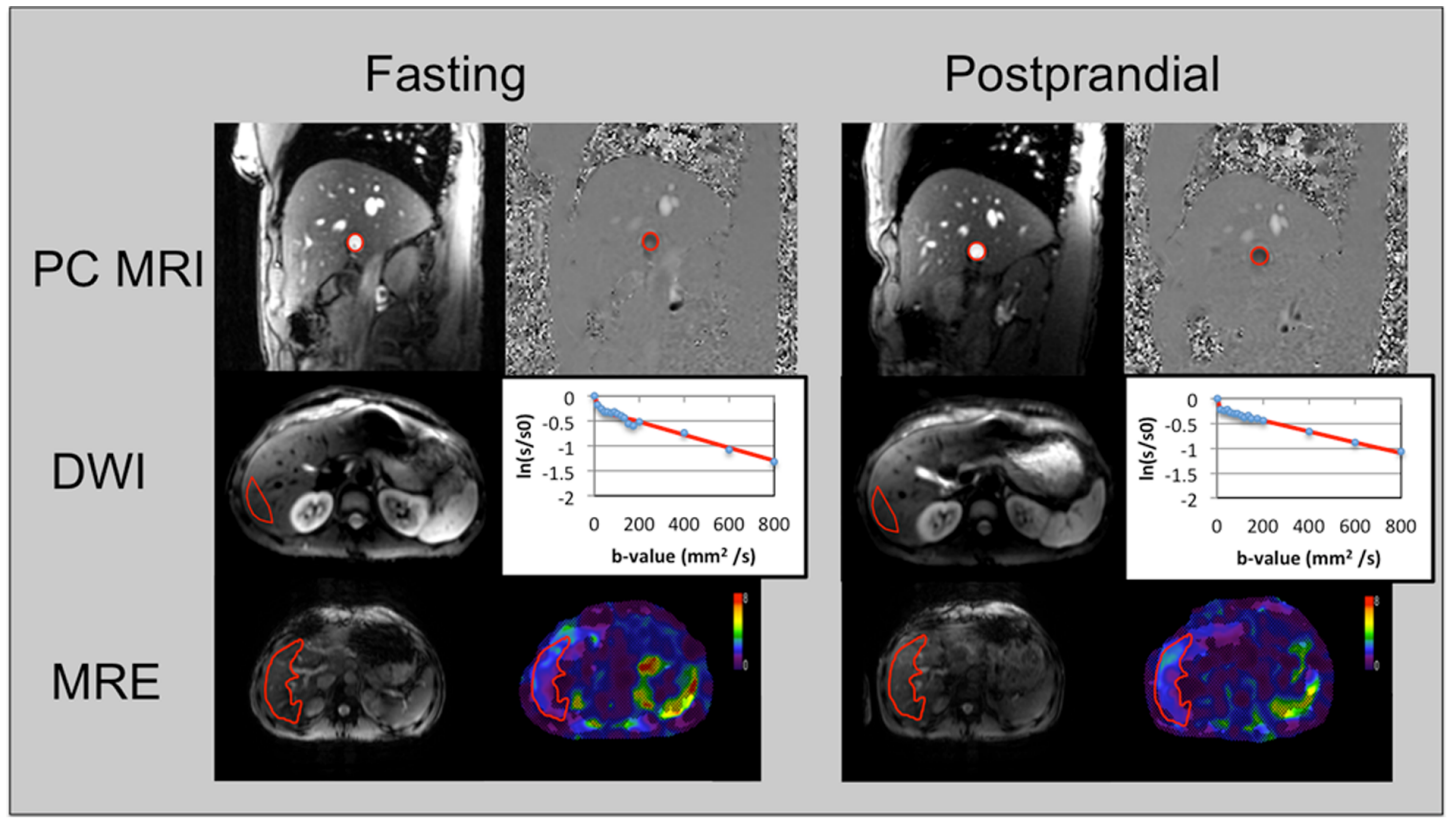
Image processing demonstrated in a 30 year-old healthy volunteer. Top row demonstrates the region of interest placement in the portal vein (PV) on the phase contrast magnitude and phase images obtained in fasting and postprandial conditions. PV flow and velocity values were 21.15 ml/s/2.7 cm/s (fasting) and 31.1 ml/s/17.8 cm/s (postprandial). Middle row demonstrates the region of interest placement in the right hepatic lobe on the diffusion images (for b = 15) and plots for bi-exponential fitting in fasting (true diffusion coefficient D, pseudodiffusion coefficient D* and perfusion fraction PF of 1.3×10^−3^ mm^2^/s, 57×10^−3^ mm^2^/s and 22%) and postprandial conditions (1.1×10^−3^ mm^2^/s, 171×10^−3^ mm^2^/s, 19%). Bottom row shows the gradient echo MRE reference image (left) and liver stiffness (LS) maps in fasting and postprandial conditions (LS was 1.9 and 1.9 kPa in fasting and postprandial conditions, respectively).

Phase contrast images were processed in order to quantify PV flow and velocity. A ROI was drawn over the PV lumen on the phase image **(** **Fig. 2** **)**. The mean velocity of the ROI was extracted for each one of the 25 phase images, and the time average was computed. The average PV area was extracted, and PV flow was computed as the multiplication of area and velocity.

#### IVIM DWI

A ROI with mean surface of 61.9±23.8 cm^2^ was placed in the right hepatic lobe in order to measure mean signal intensity (SI) **(** **Fig. 2** **)**. The left lobe was avoided due to possible cardiac motion induced artifacts. Large vessels and focal lesions were also avoided. The same ROI was propagated to the images corresponding to the b values of 0 and 800 s/mm^2^. The ADC was computed by assuming a mono-exponential decay model
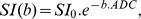
where SI(b) is the mean signal intensity in the ROI for a given b value and SI_0_ is the average signal intensity in the ROI when b = 0 s/mm^2^. A least-square method was used to estimate the value of ADC given the observed SI(b) for b = 0 s/mm^2^ and b = 800 s/mm^2^.

Liver true diffusion coefficient D, pseudo-diffusion coefficient D* and perfusion fraction PF can be estimated by assuming a bi-exponential model given by
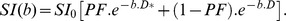



A Bayesian method was used to estimate D, D* and PF using all 16 b values, as described by us before [15].

#### MRE

LS was calculated by placing ROIs (48.4±26.2 cm^2^) on stiffness maps in the whole liver **(** **Fig. 2** **)** excluding vessels and lesions using MREQuant software (Mayo Clinic). Voxels within these ROIs with low signal-to-noise ratio (SNR) and/or with multipath wave interference provide low confidence in the computed LS and were excluded. Mean LS was obtained by averaging the LS of all remaining voxels in the ROIs.

The change in each parameter (P) when comparing the first test in fasting state with the postprandial state was computed as ΔP = 100*(P_PS_–P_FS_)/P_FS_, where P is one of the considered parameters: D, D*, PF, ADC, PV flow, PV velocity and LS. FS and PS stand for fasting state and postprandial state, respectively.

### Statistical Analysis

Matlab R2013b (The MathWorks, Inc., Natick, MA) was used for statistical analysis. A paired Wilcoxon test was performed to assess differences in PV flow, PV velocity, liver ADC, D, D* and PF, and LS between fasting and postprandial states. For each parameter that did not display a significant change postprandially, a retrospective power analysis was performed. Mann-Whitney U-tests were used to compare the same parameters between healthy volunteers and patients in both states. Short-term reproducibility in fasting state of these quantitative MRI parameters was evaluated by computing the coefficients of variation (CV) and Bland-Altman limits of agreement.

## Results

Subjects had a mean body mass index (BMI) of 25.14±4.68 kg/m^2^, with no significant difference between healthy volunteers and patients (p = 0.23). Subjects were further divided into two groups: low BMI (n = 16) vs. overweight/obese (n = 14) using a threshold BMI of 25 kg/m^2^.

Liver biopsy findings performed within 3 months of the MRI study in 8 patients demonstrated the following distribution of METAVIR fibrosis stages: stage 1 (n = 1), stage 2 (n = 1), stage 3 (n = 2) and stage 4 (n = 4). Additionally, 11 patients were diagnosed with cirrhosis based on imaging findings, 9 of which were listed for liver transplantation, and 2 had hepatocellular carcinoma.

MRE failed in 3/30 (10%) patients likely due to iron deposition (n = 2) or large volume ascites (n = 1). IVIM DWI and PC MRI did not fail in any of the subjects.

### Short-term Reproducibility of MR Metrics in Fasting Condition (Table 2)

PV flow/velocity, liver D, ADC and LS showed good to excellent short-term reproducibility in fasting state, with CVs in the range of 3.8%–15.2%. PF and D* showed acceptable and poor reproducibility, respectively (mean CVs of 20.4% and 51.6%, respectively).

**Table 2:** Short-term reproducibility of phase contrast metrics (PV flow, PV velocity), DWI metrics (liver D, D*, PF and ADC) and liver stiffness (LS) measured in 30 subjects (expressed as mean ± SD) in fasting state (measured twice).

	Fasting #1	Fasting #2	Mean CV	BA limits of agreement (%)
**PV flow**	15.8±5.4	16.4±5.5	11.5%	−35.7, 44.0
**PV velocity**	10.9±3.0	10.7±3.0	9.0%	−34.6, 30.1
**D**	1.0±0.2	1.0±0.2	15.2%	−63.5, 69.7
D*	67.9±57.3	68.9±77.3	51.6%	−200.5,184.5
**PF**	21.5±10.9	19.8±8.2	20.4%	−76.9, 69.0
**ADC**	1.3±0.2	1.3±0.3	8.2%	−30.6, 31.2
LS**	3.6±1.9	3.5±1.8	3.8%	−15.0, 11.9

CV (coefficients of variation) and Bland-Altman (BA) limits of agreement are calculated to assess reproducibility in fasting conditions.

PV: portal vein, PV flow (ml/s), PV velocity (cm/s), D (true diffusion coefficient, ×10^−3^ mm^2^/s), D* (pseudodiffusion coefficient, ×10^−3^ mm^2^/s), PF (perfusion fraction, %), ADC (apparent diffusion coefficient, ×10^−3^ mm^2^/s), LS (liver stiffness, kPa) **: LS calculated in 27 subjects.

The CVs were not significantly different between low BMI vs. overweight/obese subjects (Mann-Whitney tests, p = 0.14−0.98).

### Postprandial Changes

**Figure 3:**
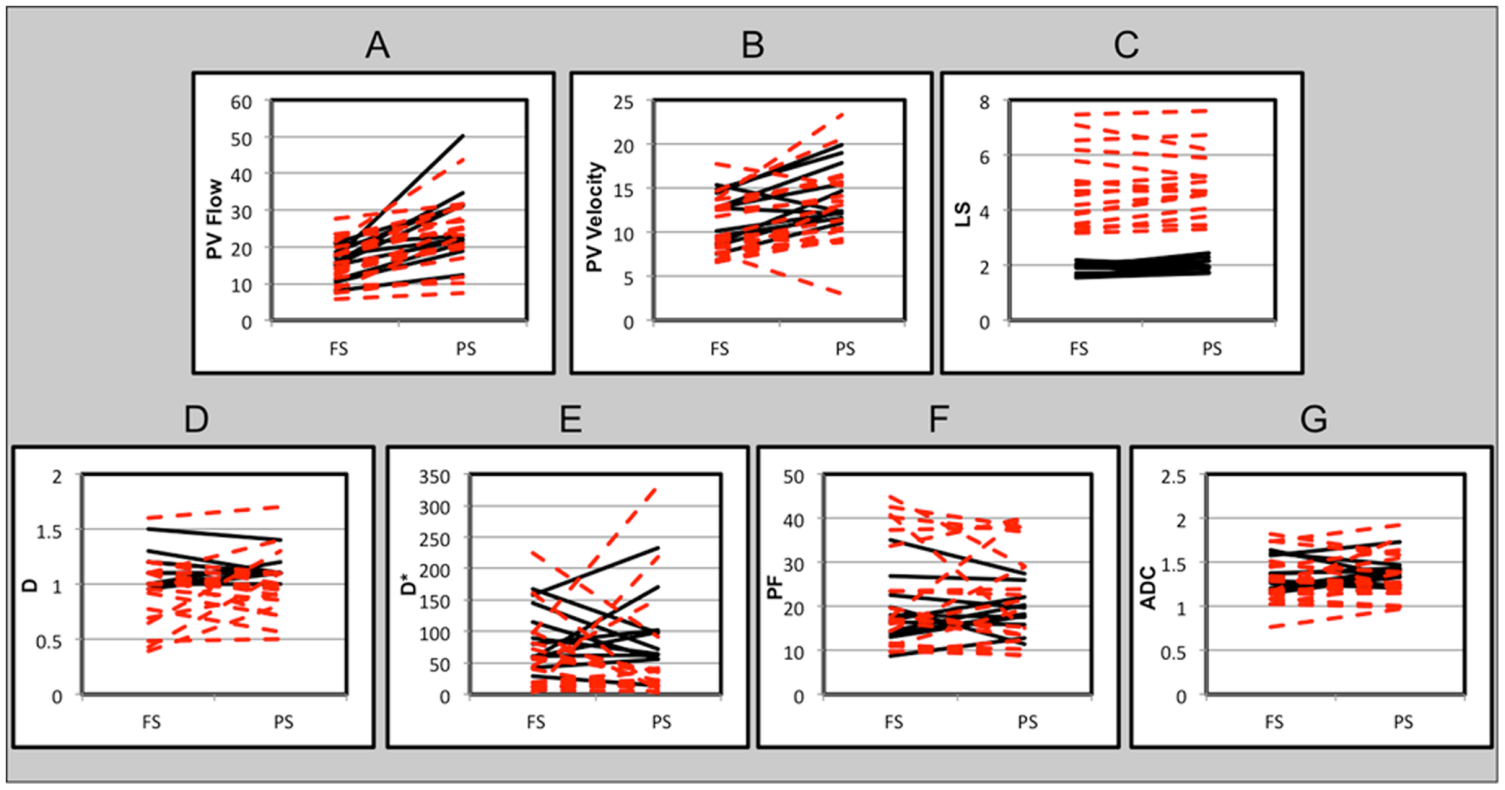
Changes in portal vein (PV) flow (A), PV velocity (B), liver stiffness LS measured with MRE (C), liver true diffusion coefficient D (D), pseudodiffusion coefficient D* (E), perfusion fraction PF (F), and apparent diffusion coefficient ADC (G) in fasting (1^st^ exam) and postprandial conditions in 11 healthy volunteers (solid black lines) and 19 patients (dashed red lines). LS shows a clear separation between healthy volunteers and patients. PV: portal vein, PV flow (ml/s), PV velocity (cm/s), LS (liver stiffness, kPa), D (true diffusion coefficient, ×10^−3^ mm^2^/s), D* (pseudodiffusion coefficient, ×10^−3^ mm^2^/s), PF (perfusion fraction, %), ADC (apparent diffusion coefficient, ×10^−3^ mm^2^/s). *LS was calculated for 27 subjects.

- As expected, PV flow and velocity were both significantly higher in postprandial state (p<0.001) **(** **Table 3** **,** **Fig. 3** **)**. PV flow did not decrease in any patient beyond the mean CV. PV velocity decreased in one patient (Δ = −13.8%) and one volunteer (Δ = −19.5%), and increased in all other subjects.
- LS was also significantly higher in postprandial state (p = 0.04). Two volunteers and four patients had a decrease in LS higher than mean CV (Δ = −10.0% and −4.0% in 2 volunteers and −12.8%, −10.0%, −8.1% and −5.0% in 4 patients). These subjects had a mean PV blood flow increase of 32.14%±30.39%. All subjects had an average ΔLS of 6.8±12.1%, 9.3%±12.6% for volunteers, and 4.5%±10.1% for patients. The increase was not significantly different between volunteers and patients (p = 0.39).
- Liver D, D*, PF and ADC did not significantly change after caloric intake (p>0.2). The power analysis determined that the postprandial differences that could have been detected with 80% power at a two-sided significance level of 5% with the current sampling size were smaller than the standard deviations of each parameter in fasting conditions (detectable differences are 0.16×10^−3^ mm^2^/s, 55.44×10^−3^ mm^2^/s, 5.66% and 0.13×10^−3^ mm^2^/s for D, D*, PF and ADC, respectively). The mean postprandial increases were ΔD = 10.1%, ΔD* = 219.3%, ΔPF = 9.1% and ΔADC = 5.3%. Changes were not significant for patients (p>0.1, mean ΔD = 15.1%, mean ΔD* = 338.2%, mean ΔPF = 9.0% and mean ΔADC = 5.7%) and for healthy volunteers (p>0.6, mean ΔD = 1.4%, mean ΔD* = 14.0%, mean ΔPF = 9.1% and mean ΔADC = 4.7%).
- Postprandial changes of MR parameters were not significantly different between low BMI vs. overweight/obese subjects (Mann-Whitney tests, p = 0.08−1.00).

**Table 3:** Postprandial changes in phase contrast metrics (PV flow, PV velocity), DWI metrics (liver D, D*, PF and ADC) and liver stiffness (LS) measured in 30 subjects (expressed in mean ± SD).

	Fasting**	Postprandial	p***	Δ**** (%)
**PV flow**	15.8±5.4	24.6±9.2	<0.0001	60.1±46.1
**PV velocity**	10.9±3.0	13.4±4.2	0.0003	25.0±29.3
**D**	1.0±0.2	1.0±0.2	0.84	10.1±41.7
D*	67.9±57.3	71.5±78.2	0.80	219.3±968.4
**PF**	21.5±10.9	21.5±9.6	0.97	9.1±41.7
**ADC**	1.3±0.2	1.4±0.2	0.23	5.3±15.9
LS*****	3.6±1.9	3.8±1.7	0.04	6.8±12.1

PV: portal vein, PV flow (ml/s), PV velocity (cm/s), D (true diffusion coefficient, ×10^−3^ mm^2^/s), D* (pseudodiffusion coefficient, ×10^−3^ mm^2^/s), PF (perfusion fraction, %), ADC (apparent diffusion coefficient, ×10^−3^ mm^2^/s), liver stiffness (liver stiffness, kPa).

**First fasting measurement.

***Paired Wilcoxon test.

****Calculated as 100*(postprandial-fasting)/fasting.

****LS calculated in 27 subjects.

### Differences between Healthy Volunteers and Patients

**Figure 4:**
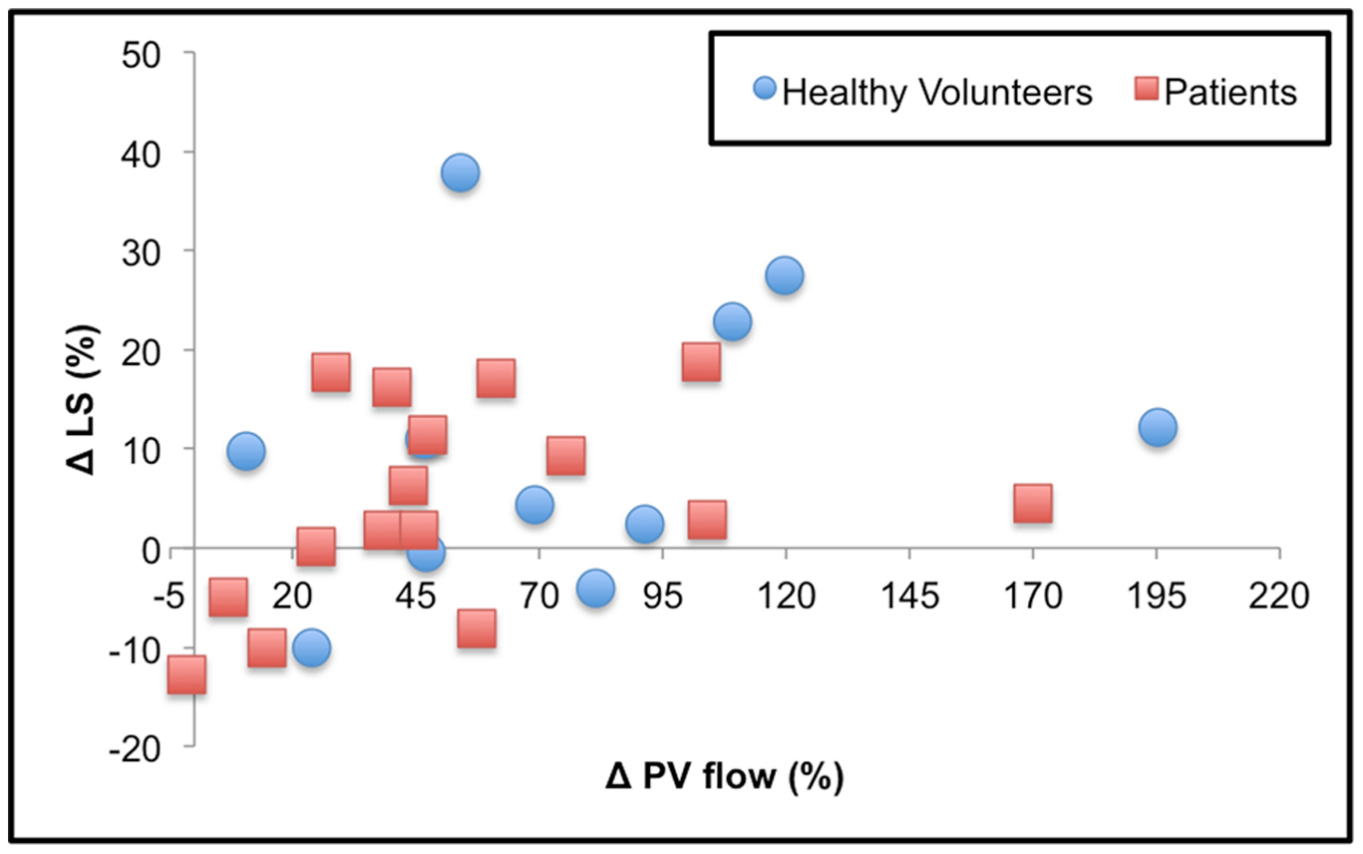
Changes in liver stiffness after a liquid meal (ΔLS*) correlated to changes in portal vein flow (ΔPV Flow*) in healthy volunteers (blue diamonds) and patients (red squares). There was a significant correlation between ΔLS vs. ΔPV Flow (Spearman rho = 0.48, p = 0.013 for all subjects; rho = 0.51 p = 0.05 for fibrosis patients, and rho = 0.41, p = 0.21 for healthy volunteers). *Δ computed as 100*(postprandial-fasting)/fasting.

- There was no significant difference in PV flow between healthy volunteers and patients (p>0.3 for both fasting and prandial states, respectively), likely due to a small sample size. Mean PV flow was 15.5±4.2 ml/s and 27.1±10.2 ml/s for healthy volunteers in fasting and postprandial states, respectively, and 16.0±6.1 ml/s and 23.2±8.5 ml/s for patients in fasting and postprandial states, respectively. There was no significant difference in PV velocity (p>0.2) as well, with mean PV velocities of 11.5±2.8 cm/s and 10.5±3.2 cm/s in fasting state for healthy volunteers and patients, respectively, and 14.4±3.2 cm/s and 12.8±4.6 cm/s in postprandial state for healthy volunteers and patients, respectively.
- Liver D* was significantly lower in patients with liver fibrosis/cirrhosis in both fasting (p = 0.03) and postprandial (p = 0.01) states **(** **Fig. 3** **)**. Liver D was significantly lower in patients only in postprandial state (p = 0.02). Liver PF and ADC showed no significant difference in either state. Mean ADC (x10^−3 ^s/mm^2^) was 1.34±0.18 and 1.39±0.14 for healthy volunteers in fasting and postprandial states, and 1.29±0.27 and 1.35±0.27 for patients in fasting and postprandial states, respectively.
- LS was significantly higher in liver disease patients compared to healthy volunteers both in fasting and postprandial states (p<0.001 for both). In healthy volunteers, the observed mean LS was 1.8±0.2 and 2.0±0.2 kPa in fasting and postprandial states, respectively, while in liver disease patients, the observed mean LS were 4.9±1.4 and 5.0±1.2 kPa in fasting and postprandial states, respectively (p<0.001 for both). There was a significant correlation between ΔLS and ΔPV Flow (Spearman rho = 0.48, p = 0.013). The correlation was stronger in patients (Spearman rho = 0.51, p = 0.05) than in volunteers (Spearman rho = 0.41, p = 0.21) **(** **Fig. 4** **)**.

## Discussion

In this study, we have quantified PV flow/velocity, LS and liver diffusion parameters twice in fasting conditions and after a liquid meal. Short-term reproducibility was assessed in fasting conditions, and the ability to differentiate between healthy volunteers and patients was also evaluated.

PV flow was shown to significantly increase between fasting and postprandial states using Doppler ultrasound and MRI in previous studies [20−26], [30], [45], . Hollingsworth et al [30] and Sadek et al [45] found a mean PV flow for healthy volunteers in the range of our study, except for the postprandial mean PV flood which was different, possibly due to a different delay of 40 minutes after caloric intake.

Hara et al [41] reported short-term reproducibility of flow measurements with PC-MRI in fasting subjects to be 11%, in agreement with our results [41]. A respiratory gating technique was used by Yzet et al [42] who reported a one year reproducibility of 17% in healthy volunteers, likely explained by the prolonged time interval.

ADC contains information on both the microcirculation of blood (perfusion) and molecular Brownian motion of water within liver parenchyma. However, the difference between fasting/non fasting in this study was not significant. This is most likely due to the fact that for the intermediate/high b values (b ≥200 s/mm^2^), ADC does not strongly depend on perfusion. Hollingsworth et al [30] observed that ADC measurements using b values of 500 and 750 s/mm^2^ in healthy volunteers were unaffected by caloric intake, while a significant change was observed when using a b value of 200 s/mm^2^. Pazahr et al [48] also reported no changes in liver ADC in the postprandial state.

Theoretically, the use of IVIM allows separating the perfusion-related coefficients (the fraction of flowing blood quantified by PF, and the velocity of capillary blood quantified by D*) from the static tissue molecular diffusion parameter D. Liver D, D* and PF did not show a significant change after a meal in our study, even though D* and PF model flow-dependent effects. This may be explained by the fact that these parameters present a limited reproducibility and differences due to the prandial state, if any, might be of the order of the parameter CV and therefore, difficult to observe. D* was shown to be significantly lower in cirrhotic patients due to reduced liver perfusion, as noted by Luciani et al [32].

In our study, we observed a concomitant increase in LS and PV flow, with a significant positive correlation, which was stronger in patients with liver disease. Changes in LS in response to a liquid meal have been observed recently using transient elastography and Doppler ultrasound [26] and MRE [40]. This correlation was not significant when only considering healthy volunteers, as observed by Hines et al [49]. Berzigotti et al [26] evaluated 19 patients with cirrhosis and portal hypertension, in whom they measured LS (using transient elastography), PV flow, and hepatic artery blood flow (using Doppler ultrasound) before and 30 minutes after a liquid meal. They observed an increase in LS (+27%±33%), which correlated with hepatic artery flow changes but not with PV flow. In 10 cases where hepatic venous pressure gradients (HVPG) were measured, postprandial hyperemia was associated with an increase in HVPG. Yin et al. [40] assessed 20 volunteers and 25 patients with fibrosis before and after a liquid meal. They observed a mean LS increase of 8.1%±10.3% in volunteers, and 21.1%±14.5% in patients (compared to 9.3%±12.6% in volunteers, and 4.5%±10.1% in patients in our study). They reported an AUC that was slightly better using postprandial LS values for differentiating volunteers from any fibrosis stage (AUC 0.97 for postprandial compared to 0.91 for preprandial LS values) [40]. While changes in LS in healthy volunteers are comparable between our study and that of Yin et al, the changes in LS in our patient population is of smaller magnitude compared to these two recent studies, possibly due to small number of patients with cirrhosis (n = 12). Therefore, despite the changes in LS values, there was no impact on the separation between volunteers and fibrosis/cirrhosis patients. In our study, any cut-off between 2.46 and 3.30 kPa would completely distinguish healthy volunteers from patients both in fasting and postprandial states. In particular, the cut-off of 2.93 kPa proposed by Yin et al [7] to detect patients with fibrosis would make no classification error. However, the optimal cut-off after a caloric intake is expected to be slightly higher than the one for the fasting state.

For 4 patients and 2 volunteers a decrease of LS was observed after the caloric intake, with a mean LS decrease of −8.35%±3.32% and mean portal venous blood flow increase of 32.14%±30.39%. A postprandial decrease of LS has been observed previously by Hines et al. [49] (4/12 volunteers had mean decrease of −9.30%±3.35%), and by Berzigotti et al. [26] (3/19 patients had mean decrease of −19.47%±15.56%). The observed postprandial decrease in the LS in our study could be partially due to the longer delay between fasting and postprandial exams, which may increase the variability in the measurements. Also, even though the ROI placements were kept as similar as possible, the confidence maps generated when computing the LS maps may slightly differ due to differences in SNR, multipath interferences and driver placements which increases the variability as well. However, we do believe that some of the observed LS decreases are beyond parameter reproducibility, and may represent postprandial biological changes and should be further investigated.

One limitation of this study is the small sample size and small number of patients with cirrhosis, which precludes the evaluation of the diagnosis power for staging hepatic fibrosis, which was not the objective of the study. The influence of BMI in the measured parameters should also be tested in a larger study.

In conclusion, this study demonstrated: a) a good to excellent reproducibility of PV flow/velocity measured with PC MRI, diffusion (D/ADC) and LS in fasting conditions; b) a significant increase in PV flow and LS after a liquid meal; c) no effect of caloric intake on IVIM diffusion parameters. We suggest that patients should undergo MR examination in a controlled fasting state when PC MRI and/or MRE are performed.
